# Optimal specimen type for accurate diagnosis of infectious peripheral pulmonary lesions by mNGS

**DOI:** 10.1186/s12890-020-01298-1

**Published:** 2020-10-15

**Authors:** Qing Wang, Bo Wu, Donglin Yang, Chao Yang, Zhixian Jin, Jie Cao, Jing Feng

**Affiliations:** 1 Department of Respiratory and Critical Care Medicine, Tianjin Medical University General Hospital, Tianjin, 300052 China; 2 Respiratory Department of Kunming Municipal First People’s Hospital, Kunming, 650000 China; 3 Transplantation Center, Nanjing Medical University, Affiliated Wuxi People’s Hospital, Wuxi, 214023 China; 4 Hematopoietic Stem Cell Transplantation Center, Institute of Hematology and Blood Disease Hospital, Chinese Academy of Medical Sciences and Peking Union Medical College, Tianjin, 300052 China

**Keywords:** Infectious peripheral pulmonary lesions, Metagenomic next-generation sequencing (mNGS), Transbronchial lung biopsy (TBLB), Bronchoalveolar lavage fluid (BALF), And bronchial needle brushing

## Abstract

**Background:**

Reports on the application of metagenomic next-generation sequencing (mNGS) to the diagnosis of peripheral pulmonary lesions (PPLs) are scarce. There have been no studies investigating the optimal specimen type for mNGS.

**Methods:**

We used mNGS to detect pathogens in matched transbronchial lung biopsy (TBLB), bronchoalveolar lavage fluid (BALF), and bronchial needle brushing (BB) specimens from 39 patients suspected of having infectious PPLs. We explored differences in microbial composition and diagnostic accuracy of mNGS for the 3 specimen types.

**Results:**

mNGS was more sensitive than conventional culture for detection of bacteria and fungi in TBLB, BALF, and BB specimens, with no difference in the sensitivity of mNGS across the different specimen types. mNGS showed higher sensitivity for fungi or uncategorized pulmonary pathogens in TBLB+BALF+BB compared to TBLB but not BALF or BB specimens. There were no significant differences between the 3 specimen types in the relative abundance of pathogens, or between TBLB and BB specimens in the relative abundance of 6 common lower respiratory tract commensals.

**Conclusions:**

mNGS has a higher sensitivity than the conventional culture method for detecting pathogens in TBLB, BALF, or BB specimens. mNGS of BB samples is a less invasive alternative to TBLB for the diagnosis of infectious PPLs.

## Background

Infectious diseases are the most common cause of death worldwide [1]. Pulmonary infections result in more deaths each year than any other type of infectious disease [2]. Delayed identification of the causative pathogen is the major cause of treatment failure and death in cases of pulmonary infection. Current microbiological tests such as culture-based methods have limitations in terms of sensitivity [3, 4], speed, and the spectrum of available assay targets [5]. Failure to identify the etiologic agent can lead to nonspecific and ineffective antibiotic therapy, resulting in adverse outcomes [5, 6]. Timely identification of infectious pathogens allows tailoring of antimicrobial regimens, which can improve the prognosis of pulmonary infections.

Advances in genomic sequencing technologies and bioinformatics approaches have provided useful tools for clinical diagnostics such as metagenomic next-generation sequencing (mNGS) [7–9]. mNGS is an unbiased approach that can in theory detect any microorganism in a clinical sample [10], and can distinguish etiologic microorganisms from background commensals [11]. Several studies have used mNGS to detect pathogens in infections of the central nervous system [12–14], digestive tract [15, 16], systemic circulation [17, 18], and lungs [19–23]. However, there have been few reports on the use of mNGS for diagnosing pulmonary infections, and the types of sample tested were mainly limited to percutaneous lung biopsy [19] and bronchoalveolar lavage fluid (BALF) [20–23] specimens.

Peripheral pulmonary lesions (PPLs), which are lesions surrounded by normal lung parenchyma, are difficult to visualize by bronchoscopy and thus pose a challenge for diagnosis. Computed tomography (CT)-guided percutaneous needle biopsy has a high diagnostic yield for PPLs, but is invasive and has a relatively high incidence of complications such as bleeding or pneumothorax [24, 25]. Bronchoscopy is appropriate for initial investigation of PPLs because of the lower complication rate [26]. Most previous studies have focused on the use of bronchoscopy in diagnosing malignancies, and there is little information on its application to the diagnosis of infectious PPLs.

The present study comprised 2 parts. We first retrospectively evaluated the performance of mNGS for detecting pathogenic microorganisms in infectious PPL samples obtained by ultrathin bronchoscopy in conjunction with virtual bronchoscopic navigation (VBN) and rapid on-site cytological evaluation (ROSE), and compared the diagnostic accuracy of mNGS with that of the conventional culture method. We also analyzed the microbial composition of transbronchial lung biopsy (TBLB), bronchial needle brushing (BB), and BALF specimens and compared the diagnostic accuracy of mNGS for the 3 specimen types.

## Methods

### Patient selection

We retrospectively analyzed the medical records of consecutive patients who were clinically and radiologically suspected of having infectious PPLs and underwent VBN-assisted ultrathin bronchoscopy at our hospital between July 2018 and July 2019. Patients for whom TBLB, BB, and BALF samples were not simultaneously available for mNGS as well as those with incomplete clinical data were excluded. Ultimately, 39 patients were included in the analysis.

### Specimen collection and processing

All bronchoscopies were performed by the same experienced bronchoscopist. The patient was locally anesthetized with 2% lidocaine, and an ultrathin bronchoscope (BF-typ XP260F; Olympus, Tokyo, Japan) with an external diameter of 2.8 mm and channel diameter of 1.2 mm was navigated to the target bronchus as far as possible using a VBN system (Direct Path 1.0). TBLB, BB, and BALF were sequentially collected from peripheral lesions in each subject. TBLB was performed first, while ROSE was performed during the examination to determine whether the amount of sample was sufficient for analysis. BB was then performed using a protective needle brush, and BALF specimens were obtained after brushing the samples.

BALF specimens were separately sent to the clinical microbiology laboratory for culture and to a commercial laboratory for mNGS. TBLB specimens and sterile saline soaked with protective needle tip were sent to a commercial laboratory for mNGS. In addition to culture, other traditional pathogen detection methods based on invasive respiratory specimens obtained through bronchoscopy were described in detail in a previously published study in our center [27]. Gram staining, acid-fast staining, hexamine silver staining, the galactomannan antigen detection test (GM test) with BALF samples, and *Mycobacterium tuberculosis* / rifampicin resistance test (X-pert) with BALF samples were performed to identify bacteria including *M. tuberculosis* and fungi including *Pneumocystis carinii*.

### Rose

Biopsy specimens were smeared onto labeled glass slides and rapid staining was performed using Diff-Quik stain. The specimens were immediately evaluated under a light microscope by a cytotechnologist to determine whether the sample was sufficient for a provisional diagnosis and subsequent laboratory analyses. The assessment was communicated to the bronchoscopist, who terminated or modified the sampling process accordingly.

### mNGS

#### Sample processing and nucleic acid extraction

Lung biopsy specimens were cut into small pieces. BALF samples (0.5–3 ml) and the soaking solution of brush tips were collected according to standard procedures. DNA was extracted from the samples using the TIANamp Micro DNA Kit (DP316; Tiangen Biotech, Beijing, China) according to the manufacturer’s protocol.

#### Construction of DNA libraries

Single-stranded (ss) DNA libraries were constructed after DNA fragmentation, end repair, adapter ligation, denaturation into single strands, and circularization. DNA nanoballs were generated from the ssDNA by rolling circle amplification and loaded into the flow cell and sequenced on a BGISEQ-50 platform (BGI, Beijing, China).

#### Sequencing and bioinformatic analysis

High-quality sequencing data were generated by removing low-quality and short-length (< 35 bp) reads, followed by a computational subtraction of human sequences mapped to the human reference genome (hg19) by Burrows–Wheeler alignment. After removing low-complexity reads, the remaining data were classified by simultaneous alignment to 4 NCBI microbial genome databases (ftp://ftp.ncbi.nlm.nih.gov/genomes/) comprising whole genome sequences of 4061 viral taxa, 2473 bacterial genomes or scaffolds, and genomic sequences for 199 fungi related to human infection and 135 parasites associated with human diseases.

### Diagnosis of pulmonary infection

The final diagnosis was made based on clinical manifestations, imaging findings, pathogen detection by culture methods and mNGS, serologic examination results, ROSE, and histopathologic analysis, expert opinion, and treatment effect observations. Serologic examination included nucleic acid detection by PCR-based testing for common respiratory viruses (Epstein–Barr virus, human cytomegalovirus, respiratory syncytial virus, parainfluenza virus, and adenovirus), serum cryptococcal capsular polysaccharide antigen test for *Cryptococcus neoformans* detection, and *Mycoplasma pneumoniae* antibody detection.

### Statistical analysis

The sensitivity and specificity of mNGS for the diagnosis of pulmonary infections were evaluated based on the final diagnosis as the gold standard. SPSS v25.0 software (SPSS Inc., Chicago, IL, USA) was used for statistical analysis, and Prism v8 software (GraphPad, La Jolla, CA, USA) was used to plot the data. The chi-squared test was used to compare rates and analysis of variance was used to compare measurement data between groups. *P* < 0.05 was considered statistically significant.

## Results

### Patients

A total of 12 male and 27 female patients with a median age of 38 years (range: 15–77 years) were enrolled between July 2018 and July 2019; 29/39 patients (74%) were immune-impaired, and 33/39 were eventually diagnosed with pulmonary infection. In 6 cases the lung lesions were considered non-infectious. There were 9 cases (23%) of multiple infections. Ultimately, 43 pulmonary infections were diagnosed in 33 patients including 14 bacterial, 21 fungal, 7 viral, and 1 mycoplasma pneumonia cases. All patients were treated with broad-spectrum antibiotics prior to sample collection.

### Performance of conventional culture method for pathogen identification

**Fig. 1:**
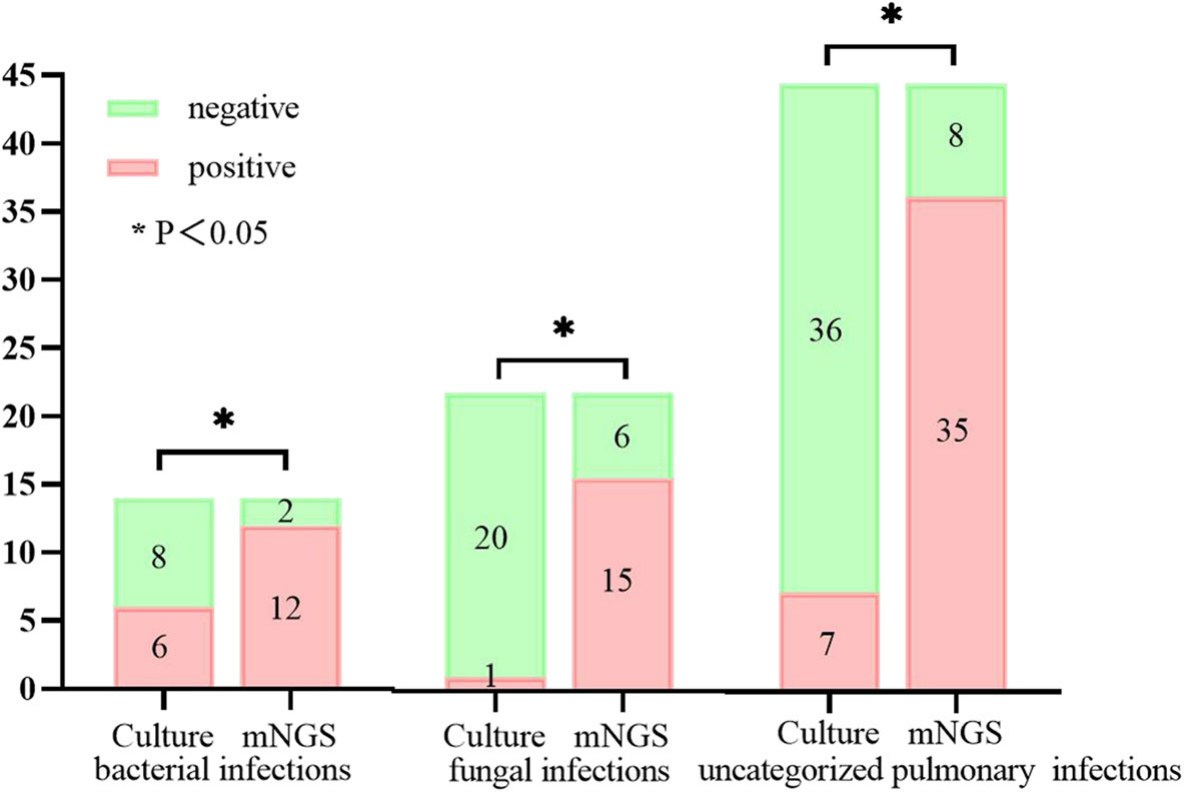
Comparison of positive rates between mNGS and microbial culture for detection of infectious bacteria, fungi, and uncategorized pulmonary pathogens. The number of positive samples by mNGS (y axis) is plotted against the mNGS and microbial cultures (x axis) of infectious bacteria (*n* = 14), fungi (*n* = 21), and uncategorized pulmonary pathogens (*n* = 43)

BALF cultures were positive in 7/43 pulmonary infections (6 bacterial and 1 fungal infection). In 5 of 6 bacterial infections detected by culture, the same bacteria were also detected by mNGS; the remaining case of *Escherichia coli* infection was missed by mNGS. Additionally, mNGS confirmed 8 bacterial infections that were missed by culture. The BALF culture was positive for *E. coli*, *Pseudomonas aeruginosa*, *Klebsiella pneumoniae*, *Corynebacterium*, and fungi. Pathogenic microorganisms detected by mNGS but not by culture were *Nocardia*, *M. tuberculosis*, *Pneumocystis jirovecii*, *Haemophilus parainfluenzae*, *Streptococcus pneumoniae*, *Pyramidobacter piscolens*, and *Prevotella*. The culture confirmed the presence of bacterial pathogens in 2 of 3 cases of *P. aeruginosa*, 2 of 2 cases of *K. pneumoniae*, 1 case of *E. coli*, and 1 case of *Corynebacterium* pneumonia. In one case, the sputum smear was positive for acid-fast bacilli and *M. tuberculosis* was detected by mNGS, but the BALF culture was negative. Thus, the sensitivity and specificity of microbial cultures for the diagnosis of pulmonary infection were 16.3 and 60.0%, respectively; and the sensitivity of microbial cultures for the diagnosis of bacterial and fungal infections was 42.9 and 4.8%, respectively (Supplemental Table 1). The positivity rates for bacteria, fungi, and uncategorized pulmonary pathogens detected by mNGS and culture tests are shown in Fig. 1. As expected, for all pathogen types mNGS was more sensitive than the conventional culture method.

### Performance of mNGS for identification of pathogens in 3 types of sample

**Fig. 2:**
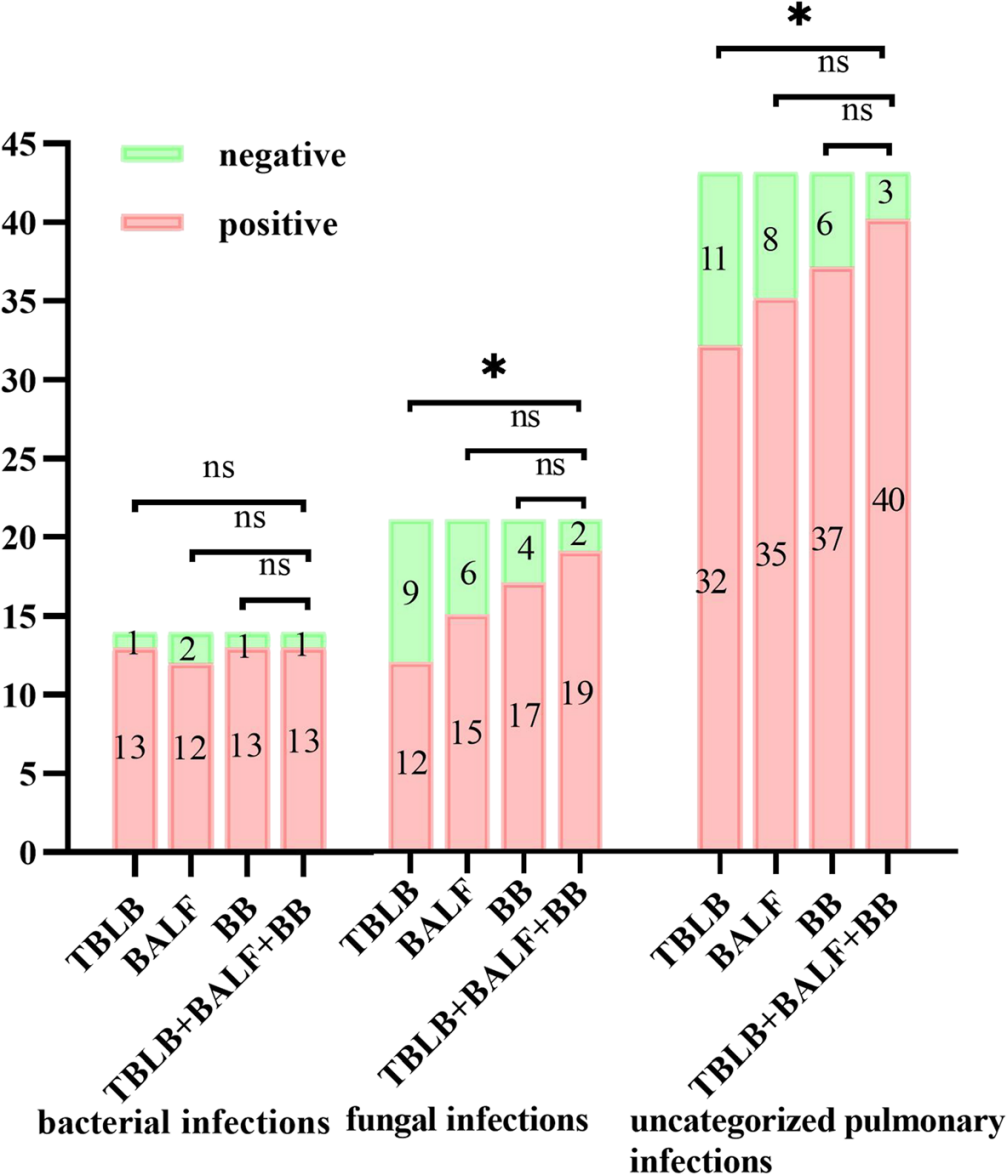
Comparison of positive rate between mNGS of TBLB, BALF, and BB samples, and mNGS of TBLB+BALF+BB for bacteria, fungi, and uncategorized pulmonary pathogens. The number of positive samples by mNGS (y axis) is plotted against TBLB, BALF, BB, and TBLB+BALF+BB groups (x axis) for infection bacteria (*n* = 14), fungi (*n* = 21), and uncategorized pulmonary pathogens (*n* = 43). ns, nonsignificant

For bacterial infections, the difference in the sensitivity of mNGS between TBLB, BALF, BB, and TBLB+BALF+BB samples was not statistically significant (Fig. 2 and Supplemental Table 1). The same was true for the detection of fungi and uncategorized pulmonary pathogens in TBLB, BALF, and BB; however, the sensitivity was significantly higher for TBLB+BALF+BB samples than for TBLB specimens. mNGS had the highest specificity in TBLB specimens, followed by BB and then BALF. However, we did not analyze differences in specificity because there were few noninfected cases, which would undermine the reliability of the results. Considering both sensitivity and specificity, our results demonstrate that BB specimens has advantages over TBLB and BALF specimens for mNGS analysis.

### Performance of mNGS and standard procedures for detecting Aspergillosis

**Fig. 3:**
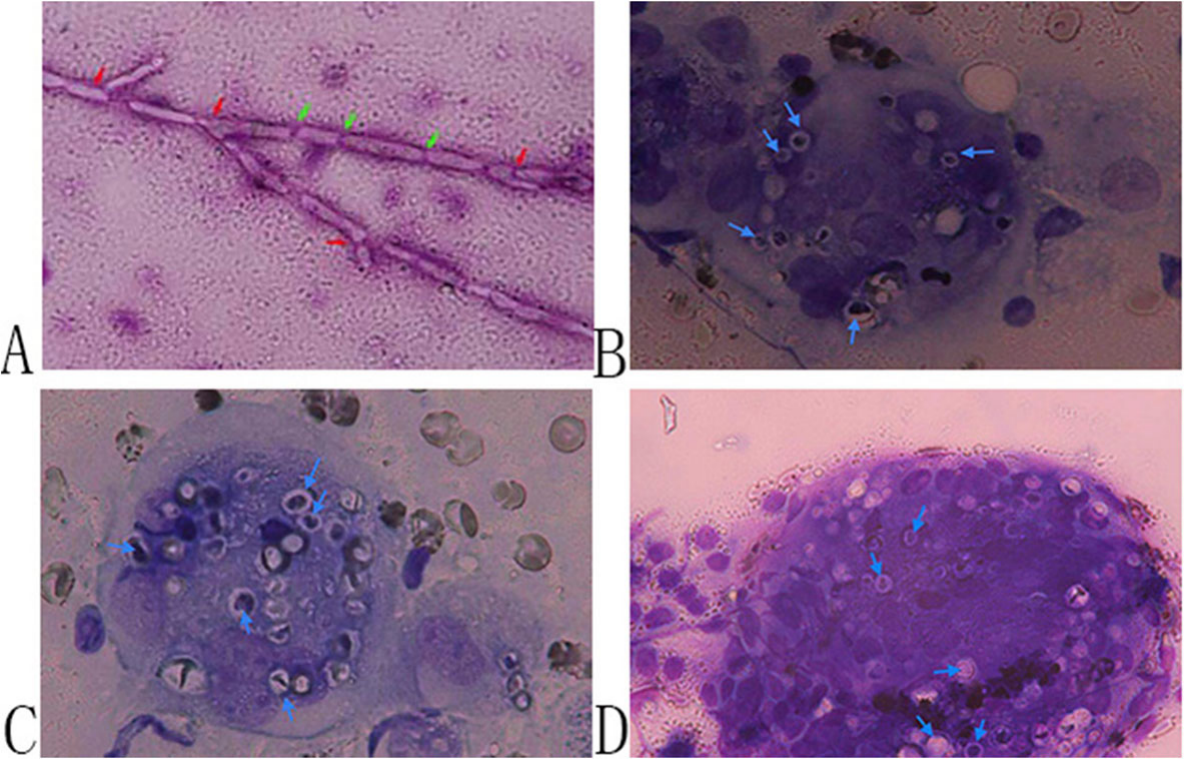
Examination of pathogens by ROSE. **a** Typical septate hyphae (green arrows) with sharp-angled bifurcations (red arrows) detected by ROSE (Diff-Quik [DQ] stain, 1000× magnification). **b–d** *Cryptococcus* (blue arrows) detected by ROSE in 3 patients (DQ stain, 1000× magnification)

Of the 21 cases of fungal pneumonia, 8 were caused by *Aspergillus*; 7 of these cases were positive by mNGS; 5 were positive by the GM test; 2 were positive by histopathologic analysis, and only 1 was positive by routine culture. One case was positive by ROSE, which revealed typical septate hyphae with sharp-angled bifurcations (Fig. 3a). Although the sensitivity of microbial culture was very low, the case missed by mNGS was detected by microbial culture, and the GM test also yielded a positive result (Supplemental Table 2).

### Performance of mNGS and standard procedures for detecting Cryptococcus

mNGS was inferior to serum antigen testing for detecting *Cryptococcus* in all patients with cryptococcal pneumonia; 1 case was missed and unidentifiable by mNGS. ROSE revealed granulomatous inflammation in all cases. *Cryptococcus* was detected by ROSE in 3 of the 4 patients (Fig. 3b–d and Supplemental Table 3).

### Characteristics of pathogens and common lower respiratory tract (LRT) commensals in different types of sample

We compared the relative abundance of pathogenic microorganisms in TBLB, BALF, and BB specimens (ratio of the number of reads of pathogenic microorganisms detected to the number of reads of the same microorganism type detected in the samples) at the species level by mNGS (Supplemental Table 4), and found that the relative abundance of pathogenic bacteria, fungi, and viruses did not differ significantly between the 3 types of sample.

We compared 6 common LRT commensals (*Prevotella*, *Neisseria*, *Streptococcus*, *Veillonella*, *Fusobacterium*, and *Rothia*) in terms of richness (total number of different genera of the 6 identified in each sample) and relative abundance at the genus level in the 3 types of sample (TBLB, BALF, and BB). Unexpectedly, we found no statistically significant differences between TBLB and BB specimens with respect to the abundance of the 6 common LRT commensals (Supplemental Table 5). The relative abundances of *Prevotella* and *Veillonella* were higher in BALF specimens than in TBLB and BB specimens, respectively (Supplemental Table 5). The total number of different genera in TBLB, BALF, and BB specimens was 2.90 (2.39–3.41), 4.28 (3.79–4.78), and 4.13 (3.56–4.70), respectively. Community richness was significantly lower in TBLB specimens than in the other 2 specimen types.

## Discussion

Conventional culture-based techniques that are employed to isolate pulmonary pathogens typically use selective culture media designed for specific types of microorganism and are thus biased towards those that are known; novel and rare microorganisms may therefore be missed, which explains the typically low detection rates. One study reported a sensitivity of 35.2% by the culture method in the diagnosis of infectious diseases [22]. However, another group reported that no bacterial pathogen was isolated by culture in up to 75% of pneumonia cases [4], which is similar to the culture-negative rate in another study [3]. In the present work, the culture-positive rate for the diagnosis of lung infections was 16.3%. This low rate may be explained by the fact that the patients were treated with broad-spectrum antibiotics prior to sample collection, which could affect culture results without eradicating the infection [23, 28]. Additionally, 74% of patients in our cohort were immune-impaired, and may therefore have harbored microorganisms that were fastidious or non-cultivable [3, 29, 30].

mNGS allows for unbiased detection of virtually any pathogen present in a sample through direct sequencing of extracted DNA [31, 32]. Several studies have reported the application of mNGS to the diagnosis of pulmonary infections. Different standards were used to calculate the sensitivity of mNGS for detecting pathogens in these studies, with some based on traditional methods [19–21] and others based on the final diagnosis [22, 23]. However, all of the studies concluded that mNGS has greater sensitivity and hence, an advantage over traditional culture-based methods in the identification of pathogens. mNGS has been used to detect pathogens in CT-guided lung tissue puncture biopsies; the sensitivity and specificity were 100.0% and 76.5%, respectively, for bacteria and 57.1% and 61.5%, respectively, for fungi, which were higher than the culture method [19]. The sensitivity of mNGS for detecting respiratory microbes (human metapneumovirus, respiratory syncytial virus, *Stenotrophomonas maltophilia*, human herpesvirus 6, and cytomegalovirus) in BALF from hematopoietic cell transplant patients with acute respiratory illnesses was reported to be 100%, which is higher than the rate by culture-based methods [20]. In 13 cases of pneumocystis pneumonia, *P. jirovecii* was detected in all BALF, sputum, and blood samples by mNGS and in 5/13 samples by conventional methods, demonstrating the superior sensitivity of the former approach [21]. mNGS of BALF was used to diagnose community-acquired pneumonia in immune-impaired patients; pathogens were identified in 6/13 samples by standard procedures and in 12/13 samples by mNGS [33]. Another study reported a sensitivity of 50.7% for the diagnosis of infectious disease by mNGS; however, the analyzed samples were not limited to lung specimens as they were obtained from patients with various types of infectious disease [22]. In the present study, the final diagnosis of each case was used to evaluate the sensitivity and specificity of the diagnostic method. We found that mNGS had higher sensitivity for detecting pathogens in BALF than standard cultures regardless of the type of pathogen (bacteria, 85.7% vs 42.9%; fungi, 71.4 % vs 4.8%; uncategorized pulmonary pathogens, 81.4% vs 16.3%).

mNGS was especially useful for detecting fungi in our study: fungal pneumonia was identified in only 1/21 samples by the culture method but in 19/21 samples by mNGS. The 2 patients missed by mNGS included one with cryptococcal pneumonia and one with aspergillus pneumonia; in the latter, the culture and GM test yielded positive results. These results suggest that although the culture-positive rate is low, it is important to combine culture, GM test, and mNGS for the diagnosis of fungal pneumonia to avoid missing a positive sample. Moreover, it should be noted that mNGS is not infallible; for example, it has no advantage for the diagnosis of cryptococcal pneumonia. As demonstrated in the present study, all 4 cases of cryptococcal pneumonia were positive for capsular polysaccharide antigen, but 1 case was missed by mNGS. ROSE also played an important role in the diagnosis of cryptococcal pneumonia: granulomas were found in TBLB specimens from all 4 patients and *Cryptococcus* was detected in TBLB specimens from 3 of the 4 patients. Therefore, our recommendation for the diagnosis of cryptococcal pneumonia is to test for capsular polysaccharide antigen, while mNGS is unnecessary if cryptococcal pneumonia is highly suspected based on the patient’s exposure history, clinical manifestations, imaging findings, and ROSE results.

In terms of detecting bacteria, mNGS still has advantages over culture, although this advantage is not as prominent in fungal detection. *P. aeruginosa*, *K. pneumoniae*, and *E. coli* were readily detected by culture.; however, some pathogenic bacterial pathogens detected by mNGS, including *Nocardia*, *M. tuberculosis*, *P. jirovecii*, *H. parainfluenzae*, *P. piscolens*, and *Prevotellacan’t be easily detected by culture*, which are either fastidious microbes (eg, anaerobes) or require long incubation times (eg, *M. tuberculosis*). In patient 4, 2 obligate anaerobic bacteria (*P. piscolens* and *Prevotella*) were identified and in patient 25, *P. piscolens* was detected. *P. piscolens* and *Prevotella* are usually isolated from the oral cavity of patients with dental pulp disease, periodontal infection, or alveolar abscess as well as from healthy individuals, and are potential causative pathogens of pulp and periodontal diseases [34, 35]; for instance, *Prevotella* was shown to induce severe bacteremic pneumococcal pneumonia in mice [36].

In the present study, 74% patients were immune-impaired and 23% were confirmed to have mixed pulmonary infections by mNGS. In contrast, conventional culture cannot be used to identify microorganisms in cases of infection caused by a mixture of pathogens. Thus, mNGS is a more useful diagnostic tool for immune-impaired patients who are susceptible to various pathogens.

We examined flora composition in the 3 types of lung specimen (including the relative abundance of pathogens and relative abundance and richness of common LRT commensals) by mNGS. Surprisingly, we found that TBLB and BB samples had similar flora compositions except in terms of the richness of common LRT commensals, while the relative abundance and richness of common LRT commensals were higher in BALF than in TBLB specimens.

BB specimens had a higher number of true positives by mNGS than TBLB and BALF specimens and fewer false positive and false negatives than TBLB specimens, indicating that BB is more useful than TBLB for diagnosing infective PPLs irrespective of diagnostic efficacy, relative abundance of pathogenic microorganisms, and interference from common LRT commensals. The superior diagnostic performance of BB was likely due to the fact that it contained cells and microorganisms from a larger area. Thus, high-throughput sequencing of BB is an alternative sampling method in patients with infectious PPLs that are unable to tolerate the more invasive TBLB procedure, such as those with hematologic diseases characterized by thrombocytopenia or poor platelet function.

It should be emphasized that we performed bronchoscopy in strict accordance with the accepted standards and took appropriate measures to avoid contamination: for example, the bronchoscope was introduced transnasally and passed through the vocal cords without aspiration to avoid oral contamination, and protected specimen brushes were used to obtain BB specimens while the bronchoscope was navigated to the target bronchus as far as possible using the VBN system to avoid interference by bacteria from sites other than the target. Research by other investigators [22] and our own experience of using mNGS to diagnose patients who did not receive any antibiotic treatment have shown that regardless of whether broad-spectrum antibiotics are used, the detected microorganisms are usually a mixture of pathogenic microorganisms and LRT commensals. This is likely because the sequencing procedure is highly sensitive and does not depend on bacterial viability.

mNGS can provide detailed information about lung microbiota. The following criteria have been established for a positive mNGS result: 1) the relative abundance of bacteria (excluding *M. tuberculosis*) and fungi exceeds a certain value (30%) [19]; 2) the sample is positive for *M. tuberculosis* if at least one read is detected at the species or genus level [22]; and 3) if a pathogen is detected by traditional methods and the number of reads by mNGS is >50, the sample is also considered as being positive by mNGS [19]. Correctly interpreting mNGS results remains challenging; moreover, determining which microorganism is the etiologic agent in cases of infection can be difficult as there is no clear threshold for pathogenicity. Thus, mNGS must be combined with a review of clinical information to accurately diagnose infectious diseases.

This study had some limitations. Firstly, it did not include patients who had not been treated with antibiotic agents. Because those who need to be obtained three specimens (TBLB, BALF and BB) for mNGS are often complex cases with poor response to initial empirical antibiotic agents. For economic reasons, consequently, we couldn’t assess the effect of antibiotic exposure on mNGS results. Secondly, the number of patients who had mNGS in all three samples is limited due to the relatively expensive cost of mNGS sequencing. Thirdly, the study was retrospective and we did not perform statistical comparisons of the specificity of mNGS for different sample types. This is because specimens for mNGS were collected only if the clinical information, CT findings, and ROSE results suggested the presence of infectious lesions; therefore, there were few negative cases, making the calculated specificity unreliable.

## Conclusions

This is the first study to evaluate the utility of combining VBN, ultrathin bronchoscopy, ROSE, and mNGS to diagnose infective PPLs. Additionally, no other study to date has compared the diagnostic efficacy, relative abundance of pathogenic microorganisms, and interference of common LRT commensals between TBLB, BALF, and BB specimens analyzed by mNGS. Our results demonstrate that mNGS is more sensitive than conventional culture for detecting infectious bacteria, fungi, and uncategorized pulmonary pathogens in TBLB, BALF, and BB specimens, with no difference in sensitivity for the 3 types of clinical sample. mNGS showed the highest specificity with TBLB, followed by BB and BALF, although the statistical significance of the differences between the 3 specimens was not determined. TBLB and BB samples had similar flora composition although they differed in terms of species richness of common LRT commensals, while the relative abundance and richness of common LRT commensals was higher in BALF than in TBLB specimens. We also showed that mNGS has advantages for pathogen detection in cases of mixed pulmonary infections in immune-impaired patients, and is more effective for fungus than for bacteria identification. However, it should be noted that mNGS has limited utility for detecting some types of infection such as cryptococcal pneumonia.

## Supplementary information

**Additional file 1: Supplemental Table 1.** Performance of mNGS and microbial culture method in the detection of infectious pathogens. **Supplemental Table 2.** Performance of various methods for detection of *Aspergillosis.* **Supplemental Table 3.** Performance of mNGS and standard methods for detection of *Cryptococcus.* **Supplemental Table 4.** Relative abundance of pathogenic microorganisms. **Supplemental Table 5.** Relative abundance of 6 common lower respiratory tract commensals in different types of clinical specimen.

## Data Availability

The datasets generated and/or analyzed in the current study are available in NCBI SRA database (direct link: http://www.ncbi.nlm.nih.gov/bioproject/663652; accession numbers: PRJNA663652) or are available from the corresponding author on reasonable request.

## Abbreviations

BALF: Bronchoalveolar lavage fluid
BB: Bronchial needle brushing
CT: Computed tomography
GM test: Galactomannan antigen detection
LRT: Lower respiratory tract
mNGS: Metagenomic next-generation sequencing
PPL: Peripheral pulmonary lesion
ROSE: Rapid on-site cytological evaluation
TBLB: Transbronchial lung biopsy
VBN: Virtual bronchoscopic navigation
X-pert: *Mycobacterium tuberculosis*/rifampicin resistance test
