# Supplementary material for: Optimal specimen type for accurate diagnosis of infectious peripheral pulmonary lesions by mNGS
Source: BMC Pulm Med. 2020 Oct 15;20:268. doi: 10.1186/s12890-020-01298-1 (PMC7566056; doi:10.1186/s12890-020-01298-1)
Supplement: Supplementary file 1 — Additional file 1: Supplemental Table 1. Performance of mNGS and microbial culture method in the detection of infectious pathogens. Supplemental Table 2. Performance of various methods for detection of Aspergillosis. Supplemental Table 3. Performance of mNGS and standard methods for detection of Cryptococcus. Supplemental Table 4. Relative abundance of pathogenic microorganisms. Supplemental Table 5. Relative abundance of 6 common lower respiratory tract commensals in different types of clinical specimen. [file 12890_2020_1298_MOESM1_ESM.docx]

**Supplemental Table 1.** Performance of mNGS and microbial culture method in the detection of infectious pathogens

| **Test** | **Sensitivity for infectious bacteria, %** | **Sensitivity for infectious fungi, %** | **Sensitivity for pulmonary infections, %** | **Specificity for** **pulmonary infections, %** |
| --- | --- | --- | --- | --- |
| Culture with BALF | 42.9 | 4.8 | 16.3 | 60.0 |
| mNGS of TBLB | 92.9^ab^ | 57.1^bc^ | 74.4^bc^ | 100.0 |
| mNGS of BALF | 85.7^ab^ | 71.4^ab^ | 81.4^ab^ | 70.0 |
| mNGS of BB | 85.7^ab^ | 81.0^ab^ | 86.0^ab^ | 90.0 |
| mNGS of TBLB+BALF+BB | 92.9^b^ | 90.5^b^ | 93.0^b^ | 63.6 |

^a^P>0.05 vs mNGS of TBLB+BALF+BB; ^b^P<0.05 vs microbial culture with BALF; ^c^P<0.05 vs mNGS of TBLB+BALF+BB.

BALF, bronchoalveolar lavage fluid; BB, bronchial needle brushing; mNGS, metagenomic next-generation sequencing; TBLB, transbronchial lung biopsy.

**Supplemental Table 2.** Performance of various methods for detection of *Aspergillosis*

| **Patient**  **no.** | **mNGS** | **GM test (≥0.65, positive)** | **Pathologic examination** | **Culture** | **ROSE** |
| --- | --- | --- | --- | --- | --- |
| 3 | (+) | (−) | (−) | (−) | Granulomatous inflammation |
| 9 | (+) | (+) | (−) | (−) | Hyphae of *Aspergillus* detected, visible necrosis |
| 11 | (+) | (+) | (+) | (−) | Ggranulomatous and suppurative inflammation, visible necrosis |
| 18 | (+) | (+) | (−) | (−) | Suppurative inflammation and visible necrosis |
| 22 | (−) | (+) | (−) | (+) | Granulomatous and suppurative inflammation, visible necrosis |
| 26 | (+) | (−) | (+) | (−) | Visible necrosis |
| 28 | (+) | (−) | (−) | (−) | Inflammation |
| 32 | (+) | (+) | (−) | (−) | Inflammation |

(+), positive; (−), negative; GM test, Galactomannan antigen detection; mNGS, metagenomic next-generation sequencing; ROSE, rapid on-site cytological evaluation.

**Supplemental Table 3.** Performance of mNGS and standard methods for detection of *Cryptococcus*

| **Patient**  **no.** | **mNGS** | **Serum cryptococcal capsular polysaccharide antigen** | **ROSE of granuloma and multinucleated giant cells** | ***Cryptococcus* detected by ROSE** |
| --- | --- | --- | --- | --- |
| 1 | (−) | (+) | (+) | (+) |
| 7 | (+) | (+) | (+) | (−) |
| 20 | (+) | (+) | (+) | (+) |
| 24 | (+) | (+) | (+) | (+) |

(+), positive; (−), negative; mNGS, metagenomic next-generation sequencing; ROSE, rapid on-site cytological evaluation.

**Supplemental Table 4.** Relative abundance of pathogenic microorganisms

| **Specimen type analyzed by mNGS** | **Relative abundance of pathogen, mean % (95% confidence interval)** | | |
| --- | --- | --- | --- |
|  | **Bacteria** | **Fungi** | **Virus** |
| TBLB | 29.02 (11.13–46.91) | 44.50 (24.19–64.81) | 81.15 (57.82–104.48) |
| BALF | 31.64 (13.17–50.11) | 35.52 (18.38–52.66) | 82.32 (63.39–101.24) |
| BB | 23.55 (6.23–40.87) | 34.27 (16.49–52.04) | 71.48 (49.52–93.44) |

BALF, bronchoalveolar lavage fluid; BB, bronchial needle brushing; mNGS, metagenomic next-generation sequencing; TBLB, transbronchial lung biopsy.

**Supplemental Table 5.** Relative abundance of 6 common lower respiratory tract commensals in different types of clinical specimen

| **Bacterial genus** | **Relative abundance of bacteria, mean % (95% confidence interval)** | | |
| --- | --- | --- | --- |
|  | **TBLB** | **BALF** | **BB** |
| *Prevotella* | 2.48 (0.52–4.43) | 7.82 (3.71–11.92)^a^ | 4.89 (1.40–8.38) |
| *Neisseria* | 2.17 (0.44–3.90) | 5.59 (1.70–9.49) | 2.98 (0.74–5.23) |
| *Streptococcus* | 5.45 (0.66–10.24) | 10.58 (4.96–16.19) | 7.58 (1.98–13.17) |
| *Veillonella* | 1.11 (0.28–1.95) | 2.91 (0.94–4.89)^b^ | 1.07 (0.29–1.84) |
| *Fusobacterium* | 0.11 (0.01–0.23) | 0.18 (0.47–0.31) | 0.21 (0.79–0.34) |
| *Rothia* | 0.55 (0.08–1.01) | 1.99 (0.90–3.09) | 1.23 (0.19–2.64) |

^a^P<0.05 vs TBLB; ^b^P<0.05 vs BB.

BALF, bronchoalveolar lavage fluid; BB, bronchial needle brushing; TBLB, transbronchial lung biopsy.
